# P-93. Association of Pre-Culture Antimicrobial Use with Culture Tests in Patients with Vertebral Osteomyelitis

**DOI:** 10.1093/ofid/ofaf695.322

**Published:** 2026-01-11

**Authors:** Toru Naganuma, Sei Takahashi, Tetsuro Aita, Hiroaki Nakagawa, Toshihiko Takada, Sugihiro Hamaguchi

**Affiliations:** Fukushima Medical University, Fukushima, Fukushima, Japan; Fukushima Medical University, Fukushima, Fukushima, Japan; Fukushima Medical University, Fukushima, Fukushima, Japan; Fukushima Medical University, Fukushima, Fukushima, Japan; Fukushima Medical University, Fukushima, Fukushima, Japan; Fukushima Medical University, Fukushima, Fukushima, Japan

## Abstract

**Background:**

Diagnosing vertebral osteomyelitis (VO) remains challenging and frequently delayed, contributing to poor outcomes. Although clinical guidelines recommend withholding antimicrobial therapy until microbiologic confirmation is achieved, antibiotics are often initiated prior to culture sampling. This study examined the impact of pre-culture antimicrobial use on culture positivity in patients with VO.

**Figure:**
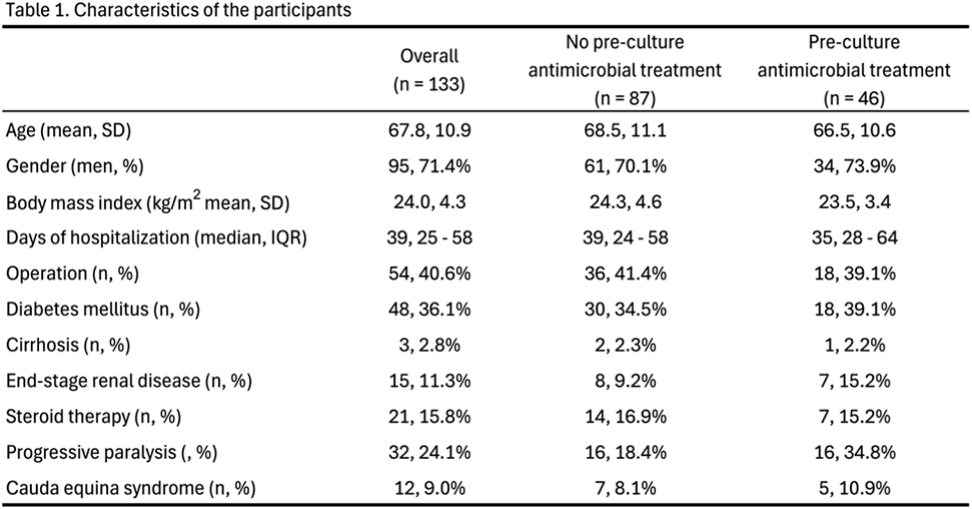

**Figure 1.**
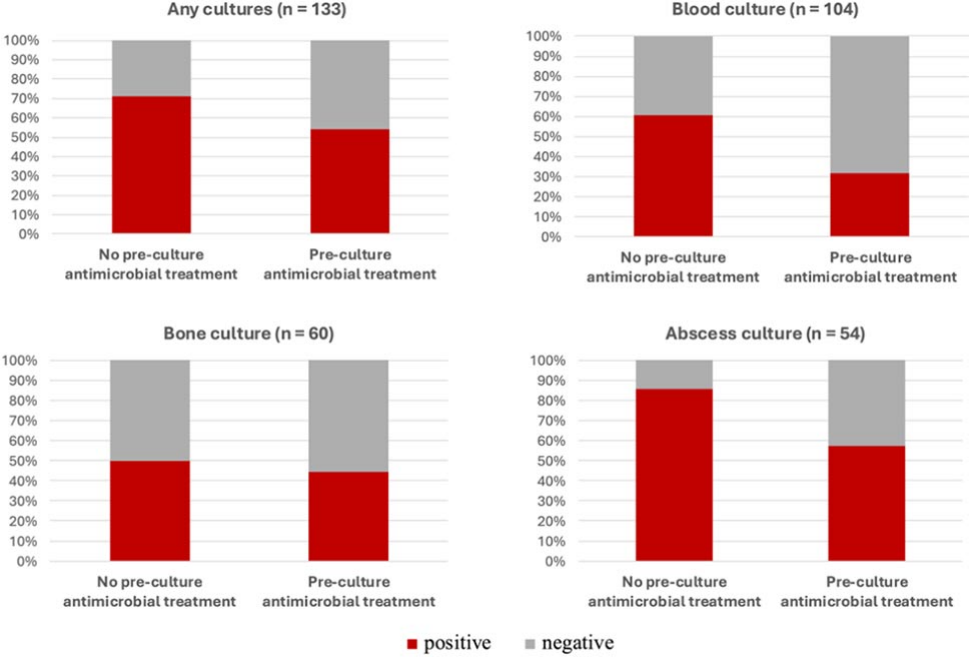
Proportions of positive culture results by pre-culture antimicrobial use status

**Methods:**

We conducted a single-center observational study using electronic medical records from a university hospital in Japan. Adult (≥ 18 years) patients admitted with a diagnosis of VO between 2006 and 2021 who underwent at least one culture test were included. Data on antimicrobial administration (start and end dates) and culture details (type: blood, bone, abscess; date; results) were extracted. Pre-culture treatment was defined as antimicrobial use within two days prior to the first culture collection. The primary outcome was the proportion of positive cultures. Logistic regression was used to estimate odds ratios (ORs), adjusting for age, sex, body mass index, diabetes, cirrhosis, end-stage renal disease, steroid use, progressive paralysis, and cauda equina syndrome.

**Results:**

Of 133 eligible patients, 46 received pre-culture antimicrobials. Baseline characteristics were similar between groups (Table 1). Blood, bone, and abscess cultures were performed in 104 (78%), 60 (45%), and 54 (41%) patients, yielding positivity proportions of 50%, 47%, and 65%, respectively. Figure 1 presents culture positivity by pre-culture treatment status. Adjusted ORs (95% confidence intervals) for culture positivity in patients with pre-culture antibiotics use were 0.31 (0.09–1.02) for blood, 0.86 (0.14–5.16) for bone, and 0.61 (0.06–6.51) for abscess cultures.

**Conclusion:**

While not statistically significant, trends toward lower culture positivity were observed with the use of pre-culture antimicrobials, particularly in blood cultures. The effect appeared less pronounced in bone cultures. Whenever feasible, culture testing should be performed prior to initiating antimicrobial therapy. However, bone and abscess cultures should still be considered even after treatment has begun, as their diagnostic yield appears less affected.

**Disclosures:**

All Authors: No reported disclosures

